# Respiratory Syncytial Virus (RSV) and Intention to Recommend RSV Vaccination: A Cross-Sectional Survey of Cardiologists and Cardiac Nurses in Southern Italy

**DOI:** 10.3390/idr16010010

**Published:** 2024-02-15

**Authors:** Domenico Ponticelli, Lorenzo Losa, Ippazio Cosimo Antonazzo, Anna Zampella, Fabio Di Marino, Gaetano Mottola, Mara Noemi Fede, Fortuna Gallucci, Roberto Magliuolo, Antonio Rainone, Antonella Arcari, Carmine Del Giudice, Pietro Ferrara

**Affiliations:** 1 Clinica Montevergine SpA, 83013 Mercogliano, Italy; 2 Center for Public Health Research, University of Milan–Bicocca, 20900 Monza, Italy; 3 Laboratory of Public Health, IRCCS Istituto Auxologico Italiano, 20165 Milan, Italy; 4 Independent Researcher, 81030 Lusciano, Italy

**Keywords:** cardiovascular disease, cross-sectional survey, elderly, respiratory syncytial virus, vaccine literacy

## Abstract

As respiratory syncytial virus (RSV) vaccine distribution gains traction in Europe and Italy, healthcare workers (HCWs) can strategize about vaccine promotion to increase uptake among patients at risk of RSV consequences, such cardiac patients. This cross-sectional survey investigated the knowledge about and attitude towards RSV and RSV vaccines, and the intention to recommend vaccination within a cardiological hospital in Italy. To explore factors associated with the outcomes of interest, multivariate logistic regression analyses were conducted. Of 197 invited HCWs, 78.2% returned the survey. The knowledge about market authorisation for new RSV vaccines for older adults (present in 46.9% of respondents) was significantly associated with the HCWs’ age, education, and previous update on vaccinations. HCWs with a higher educational level and those with a positive attitude towards RSV vaccines safety reported a higher attitude towards the importance of vaccinating people at risk. The willingness of recommending RSV vaccination to patients (70.5% of respondents) was more likely in HCWs who were knowledgeable about market authorisation for RSV vaccines and in physicians. This tempestive research sheds light on current factors influencing the strategies of cardiac HCWs regarding RSV vaccination. The results suggest the need for training events on the protective role of RSV vaccination in cardiac patients.

## 1. Introduction

Respiratory syncytial virus (RSV) stands as a significant respiratory pathogen that affects individuals of all ages, with a pronounced impact on vulnerable populations such as infants, children, and the elderly [1,2]. RSV poses a substantial risk to adults, particularly older individuals, those with underlying health conditions, and those with weakened immune systems. When infected with RSV, all these groups face an increased risk of developing lower respiratory tract disease (LRTD), which affects the lungs and can cause life-threatening pneumonia and bronchiolitis, and respiratory distress. This susceptibility is often exacerbated by age-related decline in immune function, making it challenging to mount an effective defence against the virus [3,4].

In particular, individuals with pre-existing health conditions, such as chronic respiratory diseases, heart disease, or compromised immune systems, are at an elevated risk of severe RSV-associated complications, which also include the exacerbation of chronic obstructive pulmonary disease (COPD), heart failure (HF), and asthma [5,6,7]. Research also indicates that RSV-related hospitalization is complicated by cardiovascular events in 14% to 22% of adult patients, including acute coronary syndrome and arrhythmias [8].

Every year, in Europe, 1 in 20 elderly people contracts RSV, and the infection accounts for approximately 160,000 hospitalizations, 95% of which occur in people aged 65 and older [9,10]. RSV imposes a quantifiable burden on adults in Italy, particularly among older individuals and those with underlying comorbidities [11,12]. A recent meta-analysis suggests that approximately 4.4% of respiratory samples from older adults tested positive for RSV infection [12]. Moreover, an analysis on the severity of RSV infection in older patients observed that LRTD was present in 29.5% of hospitalized patients aged 65 and above, non-invasive ventilation was implemented in 16.3%, and in-hospital death occurred in 12.1% [13]. Among others, individuals with cardiovascular disease have higher rates of healthcare utilization for RSV-associated illness and severe outcomes, and epidemiological data show that almost two-thirds of the hospitalized patients with respiratory illness due to RSV exhibit underlying cardiovascular disease [8,14,15].

Considering the above, cardiac patients represent a target population for effective measures and tools for preventing the risk associated with RSV. In fact, given the potential severity of RSV infections in these patients, as well as the general older population, there is a pressing need for preventive measures to reduce the incidence and impact of the virus. Practicing good respiratory hygiene, such as frequent handwashing and avoiding close contact with individuals displaying respiratory symptoms, can help reduce the risk of RSV transmission. In healthcare settings, stringent infection control measures are imperative to prevent outbreaks and protect vulnerable patients [16,17].

Vaccination strategies are one of the key components in safeguarding against RSV, and research has focused on developing effective vaccines for adults and the elderly. During 2023, two RSV vaccines were developed, both targeting the surface glycoprotein F (fusion) in a stabilized pre-fusion conformation (preF): an adjuvanted vaccine containing the recombinant RSVPreF3 antigen (Arexvy^®^) and a bivalent recombinant subunit vaccine that includes equal amounts of preF antigens from the two major RSV subgroups: RSV A and RSV B (Abrysvo^®^). The European (EMA) and Italian Medicines Agency (Agenzia Italiana del Farmaco, AIFA, Rome, Italy) approved both vaccines for active immunization to protect adults aged 60 years and older against LRTD caused by RSV [18,19,20,21,22,23]. More recently, in December 2023, a third mRNA-based RSV preF vaccine proved effective in reducing RSV-associated LRTD and acute respiratory disease in older adults [24,25].

Since the attainment of any vaccination programme depends on vaccine acceptance and coverage, healthcare organizations and healthcare workers (HCWs) play an essential role in improving vaccine uptake. Indeed, research shows that high coverage rates can be achieved through efforts to increase vaccine confidence, which is one of the significant barriers in the case of new vaccines [26,27,28,29]. This entails not just medical professionals but also other HCWs, such as nurses, who assume a pivotal position in promoting health and delivering health education, furnishing pertinent information to tackle the underlying reasons for non-vaccination [30,31].

To our knowledge, however, there is no exploration in the literature of HCWs’ intention to recommend RSV vaccination to at-risk patients. As mentioned earlier, attention to patients with cardiovascular conditions requires that cardiac HCWs are aware of the importance of RSV prevention, and recommending vaccination to patients should become an integral part of routine care. Therefore, the objective of this research is to assess the level of awareness regarding RSV and the new vaccination opportunity among HCWs in an Italian cardiological hospital. The goal is to shed light on possible factors that may influence HCWs’ approaches to RSV vaccines, with the ultimate aim of improving vaccination uptake and reducing the burden associated with RSV.

## 2. Materials and Methods

### 2.1. Study Design, Setting, and Population

This cross-sectional survey is part of a larger research project examining HCWs’ knowledge, attitudes, and practices of vaccination in patients with heart-related conditions and cardiovascular diseases. Here, we present the results of a survey designed to evaluate the awareness regarding RSV infection and willingness to recommend RSV vaccination among medical doctors and nurses who provide direct patient care in a cardiological hospital located in southern Italy. The facility is dedicated to diagnosing, treating, and providing care for individuals with heart-related conditions and cardiovascular diseases. It boasts specialized HCWs, state-of-the-art diagnostic instruments, and treatment options uniquely designed for cardiovascular care.

This study used a carefully selected sample by inviting all cardiologists, non-cardiologist physicians, and cardiac nurses working at the hospital where the research was conducted to participate. With the aim of achieving the best possible compliance and avoiding excessive questionnaires, the questions were administered alongside others about the recombinant zoster vaccine, which has already been the subject of publications [32]. As indicated in the previously published sister study, the assembly and determination of the sample were based on the literature on the knowledge and prevention of herpes zoster, utilizing a formula to estimate a single-population proportion, with a 95% confidence interval (95%CI) and a margin of error of 5%, which calculated a minimum sample size of 139 HCWs [32]. Participants were informed about the research’s objectives, the assurance of anonymity in data collection and usage, and the option to withdraw from the study at any time. Participation was voluntary, and no incentives were offered to HCWs.

### 2.2. Research Instrument

To examine cardiac HCWs’ knowledge, attitudes, and practices about RSV and related vaccination, an online self-administered survey was sent in November 2023 to HCWs’ work email addresses by the hospital administration, utilizing a tool specifically crafted by the IT unit of the hospital. A reminder to participate in the study was sent two weeks after the first email. The survey was anonymous, but each access from an email address was associated with a unique code to prevent response duplication. Since similar research on RSV vaccination is limited, the questions were derived by adapting knowledge, attitude, and practice surveys from the existing studies on both RSV and other vaccines [27,32,33,34]. The instrument was pre-tested and piloted with a convenience sample of 10 HCWs who closely resembled the study population. The pilot phase was conducted concurrently with that of the sister study already published [32]. Regarding the questionnaire presented here, only minor changes to the Italian wording of specific survey items were proposed and implemented.

Our survey consisted of three sections. The first was designed to collect information on HCWs’ demographic and professional characteristics, such as age, gender, professional role, educational level, hospital ward of work, experience of managing patients with RSV infection, and previous updates on vaccinations. The second section gathered information on knowledge about RSV and the RSV vaccine, attitudes towards vaccination, and the intention to encourage patients to get vaccinated as soon as the vaccine becomes fully available in Italy. The HCWs were asked about their knowledge of major complications associated with RSV, knowledge about the peak period of virus spread in Europe and Italy, awareness about the market authorisation for the RSV vaccine for the active immunisation of adults aged 60 years and older, attitudes towards the importance of vaccination for people at risk of RSV infection consequences, attitude towards RSV vaccine safety, as well as the intention to recommend or suggest RSV vaccination to patients. The third section of the survey investigated HCWs’ attitudes towards the need for further information about vaccination in special population (i.e., older adults and chronic patients), and about the new RSV vaccine, as well as their idea that patients will have concerns about the safety of the new RSV vaccines, may not accept the proposal for an ‘additional’ vaccine, or are being offered too many vaccinations.

### 2.3. Statistical Analysis

The responses were analysed using descriptive and inferential statistics. Survey characteristics were reported as frequencies and percentages for categorical variables and mean and standard deviation (SD) for continuous variables. The inferential analyses were performed in two stages, adhering to the model-building approach recommended by Hosmer et al. [35]. Initially, a univariate analysis was conducted to examine the association of independent variables with the outcomes of interest (i.e., knowledge, attitudes, and practices towards RSV), and variables with a *p*-value equal to or less than 0.25 were considered for potential inclusion in the subsequent multivariate regression models. Subsequently, according to the stepwise method for model building, only variables with a *p*-value < 0.4 on multivariate analysis were included in the final regression models.

The following three separate multivariate models were fitted to examine independent predictors of the outcomes of interest: knowledge about market authorisation for new RSV vaccines for the active immunisation of adults aged 60 years and older (Model 1); attitude towards the importance of vaccination for people at risk of RSV-associated consequences (measured on a 10-point scale; Model 2); and willingness to recommend/suggest RSV vaccination to patients (Model 3). To explore the relationship between knowledge, attitudes, and willingness to recommend/suggest the vaccine, and to examine how attitudes influence willingness, the models were developed through a step-by-step approach. This involved studying the outcomes of previous models and subsequently incorporating them as explanatory variables in the subsequent models. The variables examined for inclusion in the multivariable models are listed in the Appendix A. Those measured on ordinal scales were dichotomized before inclusion in the models. Model results were expressed as adjusted odds ratios (ORs) and 95%CIs for logistic regression (Models 1 and 3), and adjusted regression coefficients (*β*) and 95%CIs for linear regression (Model 2). A *p*-value of 0.05 was considered statistically significant. Data were analysed using Stata version 18 [36].

## 3. Results

Of a total of 197 HCWs invited to participate in the study, the survey had a response rate of 78.2%. The characteristics of the HCW population are summarized in Table 1 and have also been described in the sister publication [32]. The majority were women, the average age was 45.7 years, and 35.7% of the participants were physicians.

More than 40% of the respondents had previously taken part in a professional update on vaccinations or 35.3% reported experience in managing patients with RSV infection.

The frequency of responses regarding knowledge, attitudes, and practices towards RSV and RSV vaccines is reported in Table 2. The majority of the HCWs knew that RSV infection can cause LRTD, although fewer than half (48.7%) were aware that the RSV infection can exacerbate COPD and fewer than one-third were aware that it can lead to the worsening of HF (28.6%) or exacerbate asthma (20.8%).

In relation to the RSV vaccine, the multivariate regression analyses identified exploratory variables significantly associated with various outcomes of interest. HCWs who were aware that new RSV vaccines had received market authorization for the active immunization of adults aged 60 years and older were those who were older (OR = 1.05; 95%CI 1.01–1.09), those with the highest level of education (OR = 1.47; 95%CI 1.07–2.03), and those who had received a professional update on vaccinations (OR = 3.52; 95%CI 1.61–7.74) (Model 1; Table 3).

Measured on a 10-point Likert scale, the degree of attitudes towards the importance of vaccination for people at risk of RSV infection consequences was 7.7 (±2.0 SD), while that towards RSV vaccine safety was 7.3 (±2.0 SD). The results of the multivariable linear regression model indicated that a higher positive attitude towards the importance of RSV vaccination was observed in those with a higher educational level (*β* = 0.22; 95%CI 0.01–0.42) and in those who reported a positive attitude towards RSV safety (*β* = 2.65; 95%CI 2.15–3.16) (Model 2; Table 3).

Two variables were found to be significantly associated with the willingness to recommend/suggest RSV vaccination to patients. HCWs who were aware that new RSV vaccines had received market authorization for the active immunization of adults aged 60 years (OR = 3.82; 95%CI 1.18–12.36) and physicians (OR = 5.27; 95%CI 1.19–23.23) were more likely to express this willingness (Model 3; Table 3).

**Figure 1:**
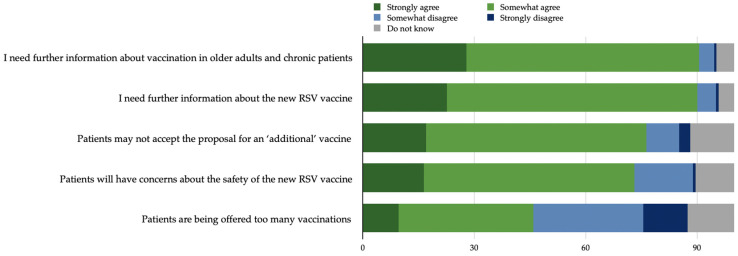
Health professionals’ attitudes (percentage of responses) regarding the need of more information about RSV vaccination and its delivery to older patients.

Lastly, the vast majority of the HCWs stated the need for more information about vaccination in older adults and chronic patients (90.5%) and about the RSV vaccine (90.1%), while 76.3% reported a belief that patients may not accept the proposal for an ‘additional’ vaccine, 73.1% that patients will have concerns about the safety of the new RSV vaccine, and 45.9% that patients are being offered too many vaccinations (Figure 1).

## 4. Discussion

During 2023, three new immunizing tools have shown encouraging results in protecting older adults from illness and death due to RSV [25]. To our knowledge, this is the first report on the assessment of a HCW population regarding their knowledge, attitudes, and willingness to recommend RSV vaccines conducted after their authorization by medicines agencies. This information is crucial especially for cardiac HCWs, who are often the central point of contact for older patients.

The first notable result is the unsatisfactory level of awareness among the surveyed HCWs regarding the consequences and complications of RSV infection. For patients with a history of cardiovascular disease, the risk of contracting this infection is associated with increased morbidity, hospitalization, and death. In fact, cardiac patients were found to experience worse clinical outcomes when affected by LRTD [37,38,39]. The same applies to the impact of RSV infection on pre-existing cardiac or respiratory conditions, such as HF, COPD, and asthma. Specifically, it is known that COPD and asthma commonly coexist with various cardiovascular diseases, exhibiting a synergistic interaction [40]. The RSV-induced exacerbations of COPD and asthma can trigger major cardiovascular events, potentially leading to severe cardiovascular outcomes and death [41,42,43].

Previous research has observed that RSV is often not diagnosed in adults with influenza-like illness [44]. This phenomenon has also been noted for other respiratory viruses, such as the influenza virus, where high rates of unrecognized cases persist [45,46]. A third of the interviewed cardiac HCWs believed that one possible reason for this underdiagnosis is the lack of specific treatment for RSV infection, as previously reported in a similar study conducted among primary care physicians in the US [33]. The reasons for investigating the aetiology of respiratory infections are diverse and go beyond the individual patient’s clinical management, extending into the realms of hygiene and public health. A concise but clear description of these reasons was provided by Talbot and Falsey to emphasize the importance of RSV and other cold viruses as a cause of infection. These authors highlighted how the identification of viral infections in adults and older adults has practical significance for isolating individuals with highly contagious viral infections during hospitalization, tailoring antiviral treatment, and reducing unnecessary antibiotic use [47].

At the time of conducting this survey, both the adjuvanted recombinant RSVPreF3 vaccine (Arexvy^®^) and the bivalent recombinant subunit vaccine (Abrysvo^®^) had received authorization from the EMA, while Arexvy^®^ also obtained authorization from AIFA (Abrysvo^®^ received it at the end of November 2023) [21,22,23]. However, reimbursement by the National Health Service (NHS) is not yet provided for either of the two vaccines. In this context, just under half of the respondents knew that a vaccine against RSV had already been authorized by EMA/AIFA and was available in Italy. Given the approval timelines and the lack of NHS coverage, this data can be considered relatively satisfactory, even though the widespread availability of the vaccine had already been communicated through various specialized channels.

However, the results underscore the need for future communication efforts by public health authorities and entities, not only directed at individuals eligible for vaccination but also, and especially, at HCWs who represent the first point of contact for patients at higher risk of sequelae from RSV infection, such as those with cardiovascular conditions. Indeed, the exploration of potential factors influencing the awareness of vaccine availability highlights participation in vaccination-focused training events as a strong predictor [48]. Topics related to preventive medicine and the prevention of communicable diseases should increasingly become an integral part of continuous education programs in clinical medicine [49]. The literature on HCWs’ behaviours towards vaccinations has already highlighted dedicated training events, contributing not only to knowledge enhancement but also to adherence to vaccination guidelines. Furthermore, as emphasized in similar surveys, the relationship between higher educational levels contributes both to a better understanding of vaccination policies and is associated with participation in ongoing medical education and staying updated on medical advancements [27,32,50]. Notably, nearly all interviewees expressed the need for more information about vaccination in special populations, as well as about the vaccination against RSV.

The analysis of cardiac HCWs’ attitudes towards the new RSV vaccination revealed that just over 50% of respondents expressed a value at the extreme end of the 10-point scale (i.e., a score equal to or greater than 8 out of 10) both regarding the importance of vaccination in their patients (59.9%) and trusting the safety of the new RSV vaccination (54.4%). The former, in particular, was associated with a higher inclination towards vaccination safety and educational level. This result underscores the significant role of training in vaccinations. More broadly, it is noteworthy that negative attitudes towards vaccines lead to hesitancy and act as major obstacles to controlling and managing preventable diseases [51,52]. This impact is even more pronounced in the case of ‘new’ vaccinations, as recently exemplified by the COVID-19 vaccine, where unfortunately, there was hesitancy among some HCWs, who cited concerns about vaccine safety among other reasons [53,54]. Regarding the importance and safety of vaccinations, every new drug undergoes rigorous quality, efficacy, and adverse effect evaluation processes before receiving market authorization by medicines agencies [55]. There is therefore an urgent need to convey messaging that emphasizes trust in vaccine safety among healthcare personnel, as well as the general population. Educating HCWs on the safety and efficacy of vaccines is crucial for improving coverage rates [32].

The beliefs of cardiac HCWs also deserve attention. A significant number of respondents agreed that patients may not accept the proposal for an ‘additional’ vaccine, may have concerns about the safety of the new RSV vaccine, and may feel that they are being offered too many vaccines. It is conceivable that these responses were influenced by common patient attitudes towards vaccinations, with common concerns leading to vaccine hesitancy [56]. This further reflects the central role of all HCWs in improving these negative perceptions among the population, especially considering the rapid development of immunization tools that can help against various communicable and non-communicable diseases. In this regard, it is essential to remember that HCWs can and should influence the willingness of patients to receive a vaccine [57].

Our results highlight an unsatisfactory level of knowledge and attitudes among cardiac HCWs regarding RSV vaccination. In a sequential process where knowledge about RSV infection and immunization tools can determine attitudes and, consequently, the behaviours of HCWs, this research can help understand the determinants that lead not all operators to declare their intention to recommend/suggest vaccination to their patients. Although 70% declare their willingness to do so as soon as the vaccine becomes fully available in healthcare facilities, there remains a high percentage of cardiac HCWs who responded negatively or expressed uncertainty. With the full implementation of these vaccinations and the potential NHS reimbursement for patients at risk of RSV-associated consequences, studying the determinants of the willingness to recommend/suggest RSV vaccination helps define timely and effective communication and training strategies for clinical staff to promote patient adherence to vaccination.

In summary, this research provides crucial information about cardiac HCWs’ knowledge, attitudes, and practices concerning RSV infection and vaccination in patients with cardiological conditions. Its added value lies in the timeliness of its conduction, concurrent with the RSV vaccines’ market authorization and before NHS reimbursement availability in Italy. The significant aspect is the opportunity to delineate critical points and determinants influencing HCWs’ practices in cardiology to address the burden associated with RSV. This will guide specific actions. Specifically, the results can be utilized in formulating educational programs aimed at clinicians to enhance their awareness of the protective role of RSV vaccination for older patients with cardiovascular conditions. Similarly, this study can serve as a reference for future research aiming to add increasingly updated information to enhance HCWs’ knowledge and practices regarding RSV vaccination. There are also broader public health implications of our work. The achieved results hold significant value in establishing guidelines cantered on preventive measures for individuals at a higher risk of RSV infection and its consequences. Disseminating these findings to public health policymakers and practitioners can indeed assist in developing shared evidence-based strategies that contribute to strengthening future RSV immunization programs, particularly in high-risk groups [58].

The main limitations of the study are as follows. First, due to its nature as a self-administered cross-sectional survey, it is possible that many responses from the surveyed HCWs could be influenced by considerations of researchers’ expectations or a tendency to conceal their actual attitudes towards certain survey items, which may therefore be subject to a social-desirability bias. However, the use of a self-administered online survey aimed to mitigate this risk. Second, as the survey was administered in a single healthcare facility, the sample of healthcare professionals may not have been representative of the broader Italian cardiac HCW population, introducing limitations to the generalizability of this research. Moreover, the sample size, although consistent with other research conducted on vaccination topics with the same population [32], may not have captured other characteristics typical of cardiac HCWs in Italy. In this sense, the limited sample size might also hinder the capacity to identify nuanced associations between population characteristics and the outcomes of interest. Third, the study was conducted before the publication, in December 2023, of the trial showing positive efficacy results for a newer mRNA-based RSV preF vaccine [24]. Therefore, the availability of this third vaccine was not considered. However, the survey considered the fact that the other two vaccines had already received market authorization before the study was conducted, and the dissemination of this authorization had already been widely publicized by the relevant authorities. Lastly, regarding the HCWs’ ultimate behaviours, it should be noted that vaccination policies and strategies in Italy may be subject to regional differences, which, at present, cannot be anticipated for a recently authorized vaccine.

To conclude, the results offer crucial insights, emphasizing the necessity for ongoing initiatives aimed at enhancing cardiac HCWs’ knowledge, attitudes, and practices concerning RSV vaccination for older patients. Support through health policies is vital to ensure accurate information, enabling HCWs to confidently recommend vaccination to their patients and effectively communicate with them to increase vaccination uptake.

## Figures and Tables

**Table 1:** Main characteristics of the study HCW population (N = 154).

Characteristic	N	Percentage
Gender		
Men	58	37.7
Women	96	62.3
Age *	45.7 ± 11.3	
Education		
Master’s or higher degree	62	40.3
Other	92	59.7
Role		
Nurse	99	64.3
Physician	55	35.7
Hospital ward		
Cardiology	18	12.0
Interventional cardiology	23	15.3
Electrophysiology	10	6.7
Cardiac surgery	21	14.0
Post-surgery intensive care	18	12.0
Cardiac intensive care	15	10.0
Cardio-pulmonology	14	9.3
More than one unit	16	10.7
Others/Non-cardiology unit	15	10.0
Previous professional update		
on vaccinations		
Yes	61	41.2
No	87	58.8
Experience in managing patients with		
RSV infection		
Yes	53	35.3
No	97	64.7

* Expressed as mean and standard deviation.

**Table 2:** Frequency of responses regarding knowledge, attitudes, and practices towards RSV and RSV vaccination.

Item *	N	Percentage
Knowledge that RSV infection can cause LRTD		
Yes	98	63.6
No	56	36.4
Knowledge that RSV infection can exacerbate COPD		
Yes	75	48.7
No	79	41.3
Knowledge that RSV infection can lead to the worsening of HF		
Yes	44	28.6
No	110	71.4
Knowledge that RSV infection can exacerbate asthma		
Yes	32	20.8
No	122	79.2
Knowledge that the peak period of virus spread in Europe and Italy is between November and March		
Yes	87	58.4
No	62	41.6
Belief that RSV infection is generally underdiagnosed because there is no specific cure		
Yes	47	32.9
No	96	67.1
Knowledge about market authorisation for the new RSV vaccines for the active immunisation of adults aged 60 years and older		
Yes	69	46.9
No	78	53.1
Attitude towards the importance of vaccination for people at risk of RSV infection consequences ^	7.7 ± 2.0	
Attitude towards RSV vaccine safety ^	7.3 ± 2.2	
Willingness to recommend/suggest RSV vaccination to patients		
Yes	103	70.5
No/Do not know	43	29.5

* Numbers for some items may not add up to the total number of the study population due to missing values. ^^^ Measured on a 10-point Likert scale. Results are expressed as mean and standard deviation. Abbreviations: RSV, respiratory syncytial virus; LRTD, lower respiratory tract disease; COPD, chronic obstructive pulmonary disease; HF, heart failure.

**Table 3:** Multivariate regression models predicting the knowledge, attitude, and practice towards RSV vaccination.

Model 1: Knowledge about Market Authorisation for New RSV Vaccines For the Active Immunisation of Adults Aged 60 Years and Older (N = 139)			
Variable	Odds Ratio	95%CI	*p*-value
Log likelihood = −80.34; *χ*^2^ = 31.14 (5 df); *p*-value < 0.0001			
Professional update on vaccinations	3.52	1.61–7.74	0.002
Age (continuous, in years)	1.05	1.01–1.09	0.02
Educational level	1.47	1.07–2.03	0.02
Knowledge of peak period of RSV spread (November–March)	1.86	0.83–4.22	0.13
Knowledge that RSV can cause LRTD	1.71	0.72–4.07	0.23
Model 2: Attitude towards the importance of vaccination for people at risk of RSV-associated consequences (N = 138)			
Variable	Coefficient	95%CI	*p*-value
F (4,133) = 35.10; R^2^ = 0.51; adjusted R^2^ = 0.50; *p*-value < 0.0001			
Positive attitude of RSV vaccine safety (≥8 vs. <8/10)	2.65	2.15–3.15	<0.001
Educational level	0.22	0.01–0.42	0.04
Knowledge that RSV can cause LRTD	0.54	−0.03–1.10	0.06
Knowledge of peak period of RSV spread (November–March)	0.24	−0.30–0.78	0.38
Model 3: Willingness to recommend/suggest RSV vaccination to patients (N = 118)			
Variable	Odds ratio	95%CI	*p*-value
Log likelihood = −35.92; *χ*^2^ = 31.97 (4 df); *p*-value < 0.0001			
Knowledge about market authorisation for RSV vaccines	3.82	1.18–12.36	0.03
Professional role (physicians)	5.27	1.19–23.23	0.03
Positive attitude towards the importance of RSV vaccination (≥8 vs. <8/10)	4.67	0.90–24.01	0.07
Belief that patients are offered too many vaccines	0.40	0.11–1.41	0.15
Positive attitude of RSV vaccine safety (≥8 vs. <8/10)	2.79	0.51–15.20	0.24
Perception that patients will have concerns about the safety of the new RSV vaccines	2.42	0.43–13.79	0.32

Abbreviations: 95%CI, 95% confidence interval; df, degrees of freedom; RSV, respiratory syncytial virus; LRTD, lower respiratory tract disease.

## Data Availability

Data and supporting materials associated with this study will be provided upon request by contacting the corresponding author.

## Appendix A. Appendix to Statistical Analysis

In Model 1: Knowledge about market authorisation for new RSV vaccines for the active immunisation of adults aged 60 years and older (Table 3), the following independent variables were examined for inclusion: gender (women = 1; men = 0), age (continuous, in years), educational level (Master’s degree or higher = 1; other = 0), professional role (physician = 1; nurse = 0), having participated to professional update on vaccinations (yes = 1; no = 0), experience in managing patients with RSV (yes = 1; no = 0), knowledge that RSV infection can cause LRTD (yes = 1; no = 0), knowledge of major RSV-associated consequences and complications (e.g., exacerbation of COPD and asthma, worsening of HF; yes = 1; no = 0), knowledge that the peak period of virus spread in Europe and Italy is between November and March (yes = 1; no = 0), belief that RSV infection is often underdiagnosed because there is no specific cure (yes = 1; no = 0), need for further information about vaccination in older adults and chronic patients (yes = 1; no = 0), and need for further information about RSV vaccines (yes = 1; no = 0).

In Model 2: Attitude towards the importance of vaccination for people at risk of RSV-associated consequences (measured on a 10-point scale; Table 3), the following independent variables were examined for inclusion: gender (women = 1; men = 0), age (continuous, in years), educational level (Master’s degree or higher = 1; other = 0), professional role (physician = 1; nurse = 0), having participated to professional update on vaccinations (yes = 1; no = 0), experience in managing patients with RSV (yes = 1; no = 0), knowledge that RSV infection can cause LRTD (yes = 1; no = 0), knowledge of major RSV-associated consequences and complications (e.g., exacerbation of COPD and asthma, worsening of HF; yes = 1; no = 0), knowledge that the peak period of virus spread in Europe and Italy is between November and March (yes = 1; no = 0), belief that RSV infection is often underdiagnosed because there is no specific cure (yes = 1; no = 0), need for further information about vaccination in older adults and chronic patients (yes = 1; no = 0), and need for further information about RSV vaccines (yes = 1; no = 0), correct knowledge about market authorisation for a new RSV vaccine for the active immunisation of adults aged 60 years and older (yes = 1; no = 0), attitude towards RSV vaccines’ safety (high = 1; middle to low = 0).

In Model 3: Willingness to recommend/suggest RSV vaccination to patients (Table 3), the following independent variables were examined for inclusion: gender (women = 1; men = 0), age (continuous, in years), educational level (Master’s degree or higher = 1; other = 0), professional role (physician = 1; nurse = 0), having participated to professional update on vaccinations (yes = 1; no = 0), experience in managing patients with RSV (yes = 1; no = 0), knowledge that RSV infection can cause LRTD (yes = 1; no = 0), knowledge of major RSV-associated consequences and complications (e.g., exacerbation of COPD and asthma, worsening of HF; yes = 1; no = 0), knowledge that the peak period of virus spread in Europe and Italy is between November and March (yes = 1; no = 0), belief that RSV infection is often underdiagnosed because there is no specific cure (yes = 1; no = 0), need for further information about vaccination in older adults and chronic patients (yes = 1; no = 0), need for further information about RSV vaccines (yes = 1; no = 0), correct knowledge about market authorisation for a new RSV vaccine for the active immunisation of adults aged 60 years and older (yes = 1; no = 0), attitude towards RSV vaccines’ safety (high = 1; middle to low = 0), attitude towards the importance of vaccination for people at risk of RSV infection consequences (high = 1; middle to low = 0), perception that patients are being offered too many vaccinations (agree = 1; disagree = 0), perception that patients will have concerns about the safety of the new RSV vaccines (agree = 1; disagree = 0), and idea that they may not accept the proposal for a ‘new’ vaccine (agree = 1; disagree = 0).
